# A phase I, randomized, single-dose study evaluating the pharmacokinetic equivalence of biosimilar ABP 215 and bevacizumab in healthy adult men

**DOI:** 10.1007/s00280-017-3416-4

**Published:** 2017-09-01

**Authors:** Richard Markus, Vincent Chow, Zhiying Pan, Vladimir Hanes

**Affiliations:** Biosimilars Development, Amgen Inc., One Amgen Center Drive, Thousand Oaks, CA 91320 USA

**Keywords:** Biosimilar, Pharmacokinetics, Bevacizumab, ABP 215, Immunogenicity

## Abstract

**Purpose:**

This study compared the pharmacokinetic (PK) profiles of the proposed biosimilar ABP 215 with bevacizumab in healthy males.

**Methods:**

In this randomized, single-blind, single-dose, three-arm, parallel-group study, healthy subjects were randomized to receive ABP 215 (*n* = 68), bevacizumab (US) (*n* = 67), or bevacizumab (EU) (*n* = 67) 3 mg/kg intravenously. Primary endpoints were area under the serum concentration–time curve from time 0 extrapolated to infinity (AUC_inf_) and the maximum observed concentration (*C*_max_). Secondary endpoints included safety and immunogenicity.

**Results:**

AUC_inf_ and *C*_max_ were similar across the three groups. Geometric means ratio (GMR) for *C*_max_ and AUC_inf_, respectively, was 0.98 and 0.99 for ABP 215 versus bevacizumab (US); 1.03 and 0.96 for ABP 215 versus bevacizumab (EU); and 1.05 and 0.97 for bevacizumab (US) versus bevacizumab (EU). The 90% confidence intervals for the GMRs of AUC_inf_ and *C*_max_ were within the prespecified standard PK bioequivalence criteria of 0.80 to 1.25. The incidence of adverse events (AEs) was 47.1, 32.8, and 61.2% in the ABP 215, bevacizumab (US) and bevacizumab (EU) groups, respectively. When analyzed by investigational site, the incidence and severity of AEs were comparable in the ABP 215 and bevacizumab groups. There were no AEs leading to study discontinuation. No binding or neutralizing anti-drug anti-bodies was detected.

**Conclusions:**

This study demonstrated the PK similarity of ABP 215 to both bevacizumab (US) and bevacizumab (EU), and of bevacizumab (US) to bevacizumab (EU). Safety and tolerability were comparable between treatments and no subject developed binding or neutralizing anti-drug anti-bodies.

## Introduction

Bevacizumab is a recombinant humanized immunoglobulin G1 monoclonal anti-body that inhibits angiogenesis by binding to vascular endothelial growth factor (VEGF) and preventing its interaction with VEGF receptors on the surface of endothelial cells [1, 2]. Bevacizumab is approved for treatment of several types of cancer, including colon cancer, lung cancer, glioblastoma, and renal-cell carcinoma, where it has been shown to improve progression-free and overall survival when used alone or in combination with other cancer therapies [3, 4]. Bevacizumab was approved by the US Food & Drug Administration (FDA) (Avastin^®^; Genentech, Inc., San Francisco, USA) in 2004 and by the European Medicines Agency (EMA) (Avastin^®^; Roche Pharma AG, Grenzach-Wyhlen, Germany) in 2005 [3, 4]. Several proposed bevacizumab biosimilars are in development, some of which have completed or currently are in Phase III development [5–8]. ABP 215, a proposed bevacizumab biosimilar, was submitted for approval to the US FDA and EMA in 2016 [5, 9].

Guidance issued by the US FDA and EMA on the development of biosimilars specifies that biosimilars should be highly similar to the reference product with respect to quality attributes, notwithstanding minor differences in clinically inactive components. In addition, there should be no clinically meaningful differences with respect to safety, purity, and potency. Both agencies recommend “totality-of-evidence” and emphasize a stepwise approach to biosimilar development [10, 11]. This begins with demonstrating analytical and biofunctional similarity to the reference product. The next step involves demonstrating that the pharmacological profile, including PK and pharmacodynamic activity, is comparable between the proposed biosimilar and the reference product. The final step is demonstrating clinical similarity with respect to efficacy, safety, and immunogenicity in a sensitive population at the same approved dosage and route of administration as the reference product.

In preclinical studies, ABP 215 was shown to be highly similar to both bevacizumab (US) and bevacizumab (EU) with respect to structure and in vitro biological activity [12]. This Phase I randomized, single-blind, single-dose study was conducted to evaluate the PK similarity of ABP 215 to bevacizumab reference product sourced from the US and the EU. The objective of the study was to demonstrate that the PK profile of ABP 215 is similar to that of bevacizumab in healthy adult men after a single intravenous (IV) dose. The secondary objectives were to assess safety, tolerability, and immunogenicity.

## Materials and methods

### Ethical conduct of the study

This study was conducted in accordance with the provisions of the Declaration of Helsinki and in accordance with the US FDA Code of Federal Regulations, the International Conference on Harmonisation E6 Guidelines on Good Clinical Practice, and the provisions of the EU Clinical Trial Directives. Written informed consent was obtained from each subject at the screening visit prior to the initiation of any study-related procedures.

### Investigational product

ABP 215 was sourced from Amgen, Inc. (Thousand Oaks, CA, USA). Bevacizumab (US) was sourced from Genentech Inc. (San Francisco, CA, USA; a member of the Roche Group) and bevacizumab (EU) was sourced from Hoffmann-La Roche Inc. (Basel, Switzerland). Investigational drugs were supplied in single-use vials with 16 mL of solution containing 400 mg of investigational product (25 mg/mL). All subjects receiving ABP 215 received investigational product from a single lot manufactured in the country of study (US or EU). All subjects receiving bevacizumab (US) and bevacizumab (EU) received investigational product from a single lot sourced in each site (US and EU); different lots were used between the US and the EU.

### Study population

Healthy men, ≥18 to ≤45 years of age with body mass indices ≥18 and ≤30 kg/m^2^, were included in this study. Subjects were excluded if they had hypertension or a history of hypertension requiring medication; received any other anti-body or protein targeting VEGF or the VEGF receptor; a history of alcohol and/or substance abuse within the last 12 months prior to screening; received or were receiving any investigational drug (or was currently using an investigational device) within 30 days (US), 90 days (EU), or 5 half-lives (whichever was longer) before receiving the dose of investigational product; previously received bevacizumab or any product considered a biosimilar to bevacizumab.

### Study design

**Fig. 1:**
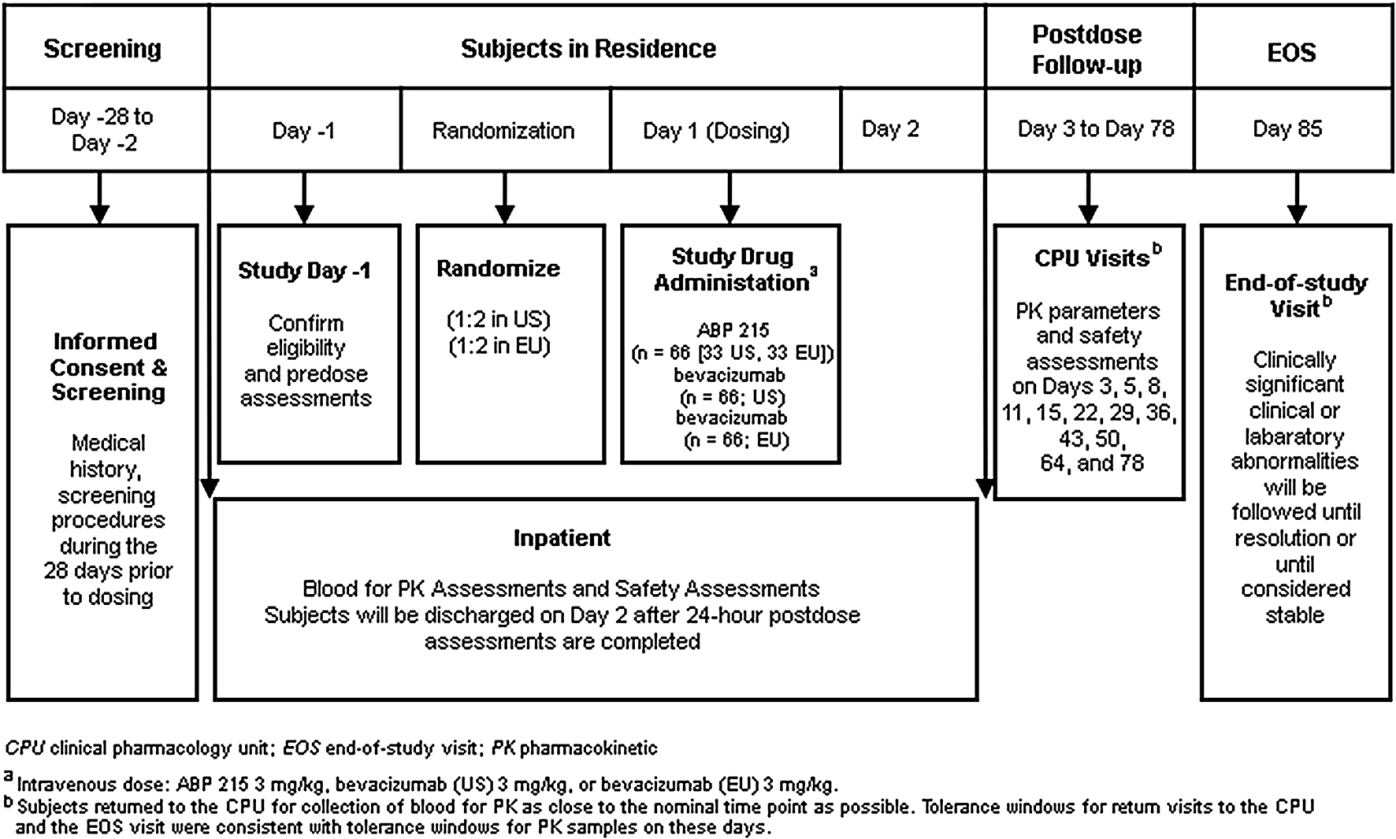
Study design

This was a randomized, single-blind, single-dose, three-arm, parallel-group study in healthy adult male subjects (Fig. 1). The study was conducted at 1 clinical pharmacology unit (CPU) located in the US and 1 CPU located in the EU. Screening occurred ≤28 days before dosing. Eligible subjects were admitted to the CPU on day 1 and randomized in a ratio of 1:2 for the 2 investigational sites such that the overall ratio of subjects receiving ABP 215 3 mg/kg IV, bevacizumab (US) 3 mg/kg IV, or bevacizumab (EU) 3 mg/kg IV was 1:1:1. PK analyses have demonstrated that bevacizumab has linear kinetics over a dose range of 1–10 mg/kg [4]; thus, the single 3-mg/kg dose was considered to be appropriate and informative across the range of therapeutic doses while minimizing drug exposure in healthy subjects.

Subjects received a single dose of ABP 215 3 mg/kg, bevacizumab (US) 3 mg/kg, or bevacizumab (EU) 3 mg/kg via IV infusion in the morning on Day 1 over 90 min. Subjects were discharged from the CPU on Day 2, and returned to the CPU on days 3, 5, 8, 11, 15, 22, 29, 36, 43, 50, 64, 78, and 85 [end-of-study (EOS) visit] for evaluation of safety, collection of PK samples, and testing for anti-drug anti-bodies (ADAs). Subjects were monitored throughout the study for AEs, clinical laboratory results, concomitant medication use, and vital signs.

### Study objectives

The primary objective of this study was to demonstrate PK bioequivalence determined by comparing AUC_inf_ and *C*_max_ in subjects treated with ABP 215 to those treated with bevacizumab (US) and bevacizumab (EU). The secondary objective was to assess the safety, tolerability, and immunogenicity in the ABP 215 group compared with those in the bevacizumab (US) and bevacizumab (EU) groups.

### PK sampling and assay

Blood samples for PK analysis were collected at pre-dose and 1.5 (end of the infusion), 4, 8, and 12 h post-dose on Day 1, at 24 h post-dose on Day 2, and at each return visit to the CPU, including the EOS visit, thereafter, up to 85 days post-dose.

A validated electrochemiluminescence (ECL) assay was used to quantify serum concentrations of ABP 215 and bevacizumab using a mouse anti-bevacizumab monoclonal anti-body (mAb) to capture the investigational product. After capturing ABP 215 or bevacizumab to the immobilized anti-body, unbound materials were removed, followed by the addition of ruthenium labeled mouse anti-bevacizumab mAb in order to detect the captured ABP 215 or bevacizumab. A tripropylamine buffer was added to enhance the ECL signals. The ECL counts were directly proportional to the amount of ABP 215 or bevacizumab bound by the capture reagent. Conversion of ECL counts to concentrations was performed using Gen5™ Secure Software v1.08.

### Anti-drug anti-bodies assay

Binding and neutralizing ADAs were detected with a two-tiered approach that included a screening assay and a confirmatory assay. Sampling for ADAs occurred on Day 1 pre-dose and at the EOS visit. A validated immunoassay was used to detect anti-bodies capable of binding ABP 215, bevacizumab (US), or bevacizumab (EU). Any sample positive for binding ADAs was to be assessed for neutralizing anti-bodies capable of binding to ABP 215, bevacizumab (US), or bevacizumab (EU) using a ligand (VEGF) binding assay.

### Primary and secondary endpoints

The primary endpoint was PK parameters (AUC_inf_ and *C*_max_). Secondary endpoints were incidence of treatment-emergent AEs (TEAEs), vital signs, laboratory safety tests, electrocardiograms (ECGs), incidence of ADAs, and AUC from time 0 to the last quantifiable concentration (AUC_last_).

### Pharmacokinetic Evaluation

PK parameters assessed included *C*_max_, AUC_inf_, *C*_max_ at last measurable serum concentration (*C*_last_), time at which *C*_max_ was observed (*t*_max_), AUC_last_, terminal elimination half-life (*t*_½_), and first-order rate constant of drug associated with the terminal portion of the curve (*λ*_z_). All values were calculated from serum bevacizumab and ABP 215 concentration data using non-compartmental methods.

### Safety

Subjects were monitored for AEs throughout the study. Vital signs measurements were taken at every CPU visit. Clinical laboratory tests (hematology, chemistry, and urinalysis) were administered at screening and at Days -1, 2, 8, 22, 43, and EOS (Day 85). Physical examinations and 12-lead ECGs were administered at screening and at Days -1, 2, and EOS. ADA assessments were performed at Day 1 (pre-dose) and EOS.

### Statistical methods

Sample size was estimated based on previous bioavailability studies with bevacizumab. Approximately, 198 subjects were to be enrolled in this three-arm study (99 per site; 66 per treatment arm).

The serum concentration versus time profile was summarized and depicted descriptively for all subjects who received any amount of investigational product and who had at least 1 reported serum concentration of bevacizumab or ABP 215. PK parameters were calculated using non-compartmental techniques (WinNonlin^®^ Professional Network Edition, Version 5.2, Pharsight Corp, St. Louis, MO) for all subjects with an evaluable bevacizumab or ABP 215 serum concentration versus time profile. The point estimate and 90% confidence intervals (CI) for ratio of the least square geometric means for *C*_max_, AUC_inf_, and AUC_last_ were estimated using an analysis of variance model. Three comparisons were performed: ABP 215 versus bevacizumab (US); ABP 215 versus bevacizumab (EU); and bevacizumab (US) versus bevacizumab (EU).

PK similarity criteria were prespecified using the standard bioequivalence margin, comparing the 90% CIs for the geometrical mean (GM) test-to-reference ratios for *C*_max_ and AUC_inf_ within 0.80 and 1.25; AUC_last_ was also evaluated to fully assess exposure to investigational product. To establish bioequivalence, the 90% CIs for the GM test-to-reference ratios for *C*_max_, AUC_inf_, and AUC_last_ had to be entirely contained within the bioequivalence margin. Prior to statistical modeling, PK parameters were log-transformed.

The safety population consisted of all subjects who received any amount of investigational product. AEs were listed by system organ class and preferred term (Medical Dictionary for Regulatory Activities, Version 15.0) and summarized by severity and relationship to treatment. In a post hoc summary, AEs were summarized by site. Clinical laboratory test, vital signs, and ECG data were summarized by time point and treatment using appropriate descriptive statistics. The number and percentage of subjects developing ADAs were tabulated for each treatment and for the overall study population.

## Results

### Subject disposition and characteristics

**Fig. 2:**
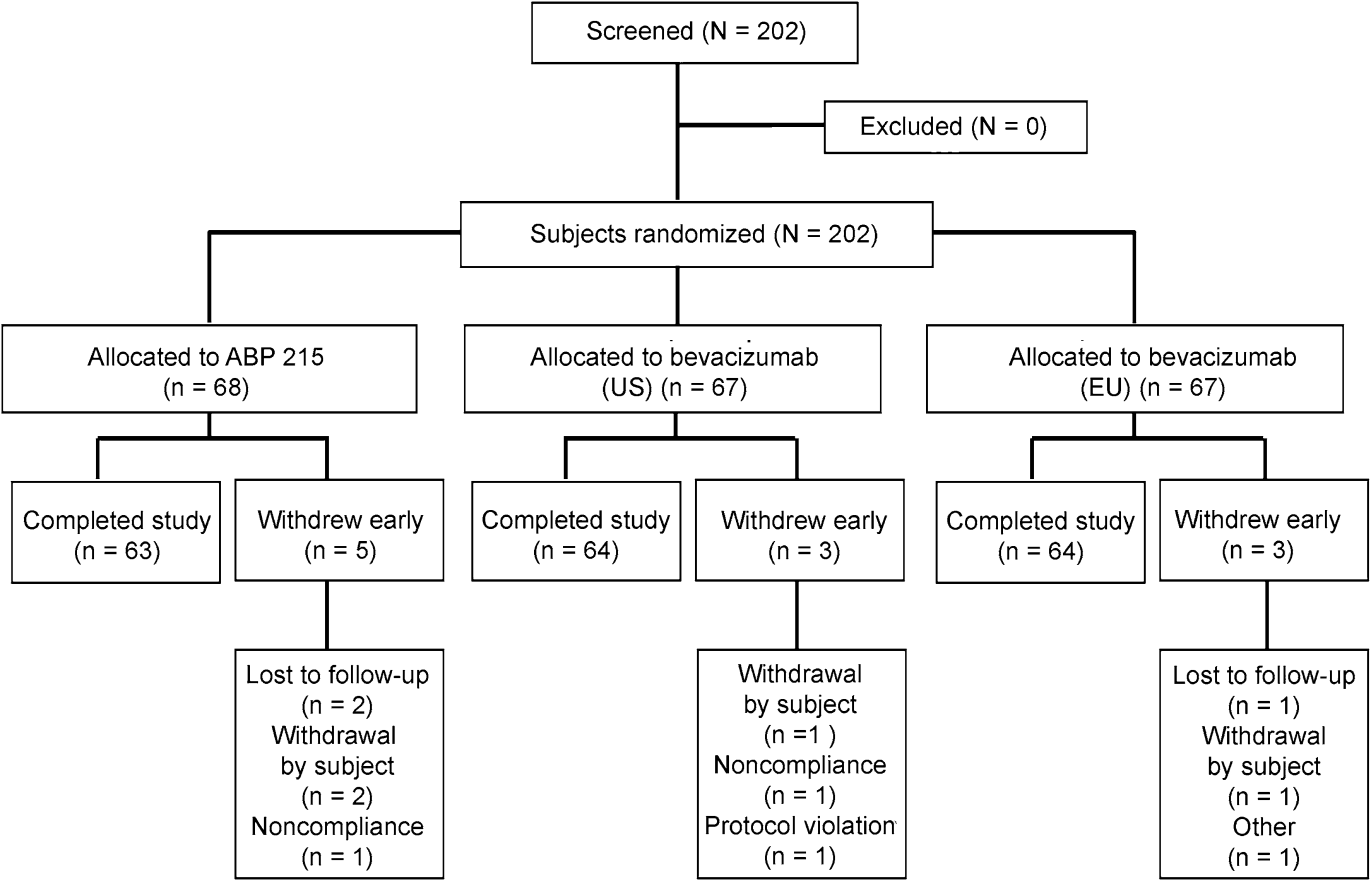
Summary of subject disposition

A total of 202 subjects were enrolled, all of whom received investigational product (Fig. 2); 68 subjects were randomized to ABP 215 (33 and 35 subjects in the US and EU, respectively); 67 subjects were randomized to each of the bevacizumab (US) and bevacizumab (EU) groups. A total of 191 (94.5%) subjects completed the study; 63 (92.6%) subjects in the ABP 215 treatment group, 64 (95.5%) subjects in the bevacizumab (US) group, and 64 (95.5%) subjects in the bevacizumab (EU) group. Eleven (5.4%) subjects discontinued from the study prematurely; 4 subjects withdrew consent [2 subjects in the ABP 215 group and 1 each in the bevacizumab (US) and bevacizumab (EU) groups]; 3 subjects were lost to follow-up (2 in the ABP 215 groups and 1 in the bevacizumab [EU] group); 2 subjects discontinued due to non-compliance [1 in the ABP 215 group and 1 in the bevacizumab (US) group]. One subject in the bevacizumab (US) group discontinued due to a protocol violation and 1 subject in the bevacizumab (EU) did not complete the study due to technical problems with the infusion pump (this subject was replaced); these 2 subjects were not included in the PK concentration, PK parameter, and per-protocol PK parameter populations. All subjects were included in the safety population.

Baseline characteristics and demographics are summarized in Table 1. Baseline characteristics were comparable between treatment groups.

**Table 1:** Baseline characteristics and demographics

Parameter	ABP 215 (*n* = 68)	Bevacizumab (US) (*n* = 67)	Bevacizumab (EU) (*n* = 67)
Mean age (years) (SD)	30.1 (7.23)	31.0 (7.02)	30.0 (7.14)
Mean weight (kg) (SD)	78.22 (9.958)	79.97 (10.387)	76.93 (10.418)
Mean height (cm) (SD)	175.2 (6.74)	175.4 (7.09)	175.9 (7.18)
Mean BMI (kg/m^2^) (SD)	25.48 (2.971)	25.99 (2.788)	24.81 (2.617)
Ethnicity, *n* (%)			
Not Hispanic or Latino	51 (75.0)	35 (52.2)	66 (98.5)
Hispanic or Latino	17 (25.0)	32 (47.8)	1 (1.5)
Race, *n* (%)			
American Indian or Alaska Native	0	2 (3.0)	0
Black or African American	17 (25.0)	14 (20.9)	13 (19.4)
Asian	10 (14.7)	0	22 (32.8)
Native Hawaiian or other Pacific Islander	0	2 (3.0)	0
White	38 (55.9)	48 (71.6)	23 (34.3)
Other	3 (4.4)	1 (1.5)	9 (13.4)

*BMI* body mass index, *N* number of subjects, *%* percentage of subjects [calculated as 100 × (number of non-missing observations/number of subjects)], *SD* standard deviation

### Pharmacokinetic results

**Fig. 3:**
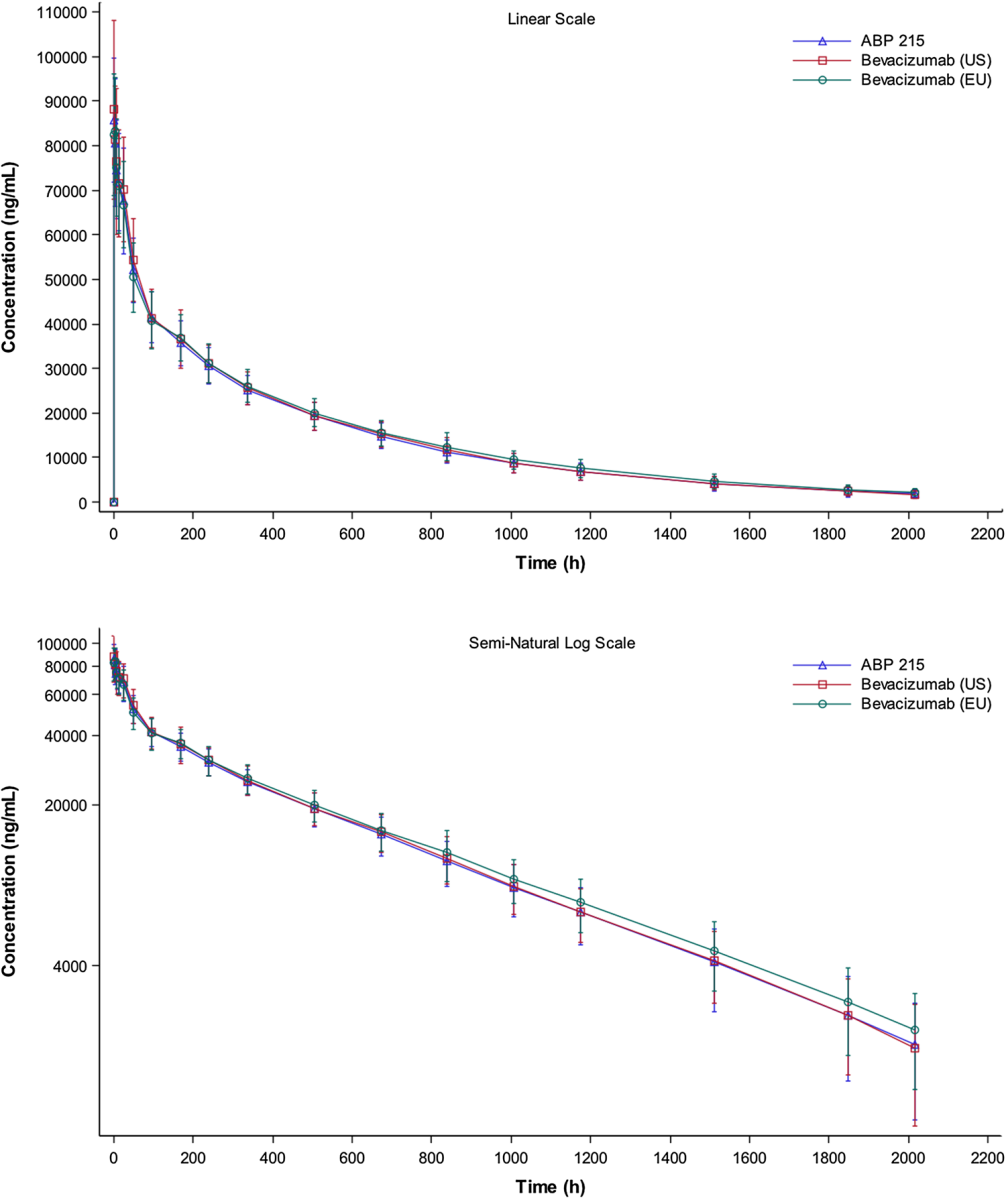
Mean (±SD) serum ABP 215, bevacizumab (EU), and bevacizumab (US) concentration; linear (*top*) and semi-logarithmic (*bottom*) scales

The mean serum concentration–time profiles were similar over the entire course of sampling following a single 3-mg/kg IV infusion of investigational product (Fig. 3). Peak concentrations were observed ~1.5–3 h after the start of the infusion, following which concentrations declined in a biphasic manner. PK parameters were similar following the single 3-mg/kg IV infusion of ABP 215, bevacizumab (US), and bevacizumab (EU) (Table 2). Both peak and overall exposure were similar across the 3 treatments, as was the *t*_max_. The terminal *t*_½_ was estimated to be, on average, 18–19 days. For the vast majority of subjects in each treatment arm, AUC_last_ accounted for at least 90% of the total AUC.

**Table 2:** Summary of pharmacokinetic parameters

Treatment	*C*_max_ (µg/mL) GM [*n*]	AUC_last_ (µg h/mL) GM [*n*]	AUC_inf_ (µg h/mL) GM [*n*]	*t*_max_ (h) median [*n*] (min–max)	*t*_½_ (days) mean [*n*] (SD)
ABP 215	87.2 [67]	28,200 [62]	29,400 [66]	1.50 [67] (1.47–24.0)	17.7 [66] (3.68)
Bevacizumab (US)	89.1 [66]	28,500 [62]	29,600 [66]	1.50 [66] (1.48–24.0)	17.5 [66] (3.39)
Bevacizumab (EU)	84.7 [64]	29,400 [64]	30,600 [66]	3.94 [64] (1.47–8.00)	18.5 [66] (3.28)

*AUC*_*inf*_ area under the serum concentration curve from time 0 extrapolated to infinity; *AUC*_*last*_ AUC from time 0 to the last quantifiable concentration; *C*_*max*_ maximum observed concentration; *GM* geometric mean; *Max* maximum; *Min* minimum; *n* number of subjects with evaluable parameters; *SD* standard deviation

PK parameters for ABP 215, bevacizumab (US), and bevacizumab (EU) are summarized in Table 2. For both ABP 215 and bevacizumab (US), the peak concentration occurred up to 24 h after the start of the 90-min infusion in a small number of subjects; despite the apparent delay, the peak concentrations were consistent with those from subjects with earlier *t*_max_.

Bioequivalence assessment of PK parameters for ABP 215, bevacizumab (US), and bevacizumab (EU) is shown in Table 3. For *C*_max_, AUC_inf_, and AUC_last_ following a single 3-mg/kg IV infusion of ABP 215 compared to bevacizumab (US) and bevacizumab (EU), the 90% CIs for the ratios of GMs were fully contained within 0.80–1.25, confirming the bioequivalence between ABP 215 and bevacizumab (US), ABP 215 and bevacizumab (EU), and bevacizumab (US) with bevacizumab (EU) (Table 3).

**Table 3:** Statistical assessment of PK parameters

Comparison	Ratio of adjusted LS geometric means (90% CI)^a^		
	*C*_max_ ratio of adjusted LS geometric means (90% CI)	AUC_inf_ ratio of adjusted LS geometric means (90% CI)	AUC_last_ adjusted LS geometric means (90% CI)
ABP 215 versus bevacizumab (US)	0.98 (0.93–1.03)	0.99 (0.95–1.04)	0.99 (0.95–1.03)
ABP 215 versus bevacizumab (EU)	1.03 (0.98–1.08)	0.96 (0.92–1.01)	0.96 (0.92–1.00)
Bevacizumab (US) versus bevacizumab (EU)	1.05 (1.00–1.10)	0.97 (0.92–1.01)	0.97 (0.93–1.02)

*AUC*_*inf*_ area under the serum concentration curve from time 0 extrapolated to infinity; *AUC*_*last*_ AUC from time 0 to the last quantifiable concentration; *CI* confidence interval; C_max_ maximum observed concentration; *LS,* least squares

^a ^For bioequivalence, the 90% CIs had to be within the bioequivalence criteria of 0.80 and 1.25

### Safety results

There were no deaths, AEs, or serious AEs (SAEs) leading to discontinuation from the study. AEs are summarized by site in Table 4. When assessed by site, the overall incidence of AEs was higher in the EU compared with the US. In the EU site, the percentage of subjects with any AE was 57.6 and 61.2% in the ABP 215 and bevacizumab (EU) groups, respectively. In the US site, the percentage of subjects with any AE was 37.1 and 32.8% in the ABP 215 and bevacizumab (US) groups, respectively.

**Table 4:** Summary of adverse events by category and investigational site

Adverse event category, *n* (%)	Investigational site			
	US		EU	
	ABP 215 (*n* = 35)	Bevacizumab (*n* = 67)	ABP 215 (*n* = 33)	Bevacizumab (*n* = 67)
Any AE	13 (37.1)	22 (32.8)	19 (57.6)	41 (61.2)
Any grade 1 AE	12 (34.3)	17 (25.4)	19 (57.6)	38 (56.7)
Any grade 2 AE	2 (5.7)	7 (10.4)	3 (9.1)	7 (10.4)
Any grade 3 AE	0	0	0	1 (1.5)
Any grade 4 AE	2 (5.7)	0	0	0
Any grade 5 AE	0	0	0	0
Any SAE	0	0	0	0
Any AE related to study drug	6 (17.1)	11 (16.4)	9 (27.3)	15 (22.4)

Subjects with multiple events in the same category were counted only once in that category. Subjects with events in more than 1 category were counted once in each of those categories

*AE* adverse event, *n* number of subjects, *SAE* serious adverse event

The incidence of AEs assessed as possibly or probably related to the study drug was 27.3% in the ABP 215 treated in the EU versus 22.4% in the bevacizumab (EU) group, and 17.1% in the ABP 215 group treated in the US versus 16.4% in the bevacizumab (US) group.

All but 3 AEs were mild to moderate (grade 1–2) in intensity; 1 subject in the bevacizumab (EU) group had an unrelated AE of eye pruritus (grade 3); 1 subject in the ABP 215 group had an unrelated AE of increased creatine kinase (grade 4); 1 subject in the ABP 215 group had an unrelated AE of exercise-induced increase in muscle enzymes (grade 4). All AEs resolved without medical intervention.

The only AE that occurred in ≥5.0% of subjects in all treatment groups was headache (Table 5). Headache was the most common AE in the bevacizumab (US) and bevacizumab (EU) groups (14.9% and 23.9%, respectively), and was also the most common AE in the ABP 215 group treated in the US (11.4%). In the ABP 215 group treated in the EU, the most common AE was nasopharyngitis and vessel puncture site hematoma (12.1% for both).

**Table 5:** Adverse events reported in >5% in any treatment group by investigational site

Adverse event, *n* (%)^a^	Investigational Site			
	US		EU	
	ABP 215 (*n* = 35)	Bevacizumab (*n* = 67)	ABP 215 (*n* = 33)	Bevacizumab (*n* = 67)
Headache	4 (11.4)	10 (14.9)	2 (6.1)	16 (23.9)
Nasopharyngitis	0	0	4 (12.1)	11 (16.4)
Vessel puncture site hematoma	0	0	4 (12.1)	1 (1.5)
Toothache	0	0	3 (9.1)	0
Dizziness	0	1 (1.5)	3 (9.1)	0
Pharyngitis	0	0	0	5 (7.5)
Acne	0	0	2 (6.1)	1 (1.5)
Nausea	1 (2.9)	4 (6.0)	1 (3.0)	1 (1.5)
Blood creatine phosphokinase increase	2 (5.7)	0	0	0
Diarrhea	2 (5.7)	1 (1.5)	1 (3.0)	1 (1.5)

^a^ By preferred term

There were no clinically relevant changes in clinical laboratory tests, ECGs, vital signs, and physical examinations. There were no pre-existing ADAs detected in the baseline samples, and no subjects had a positive ADA test at EOS.

## Discussion

Bevacizumab was approved for use in the US in 2004 and in the EU in 2005 for treatment of several types of cancer and has been shown to improve survival either alone or in combination with other cancer therapies [3, 4]. Biosimilars are expected to have minor structural differences from their reference product resulting from differences in expression systems, cell lines, bioprocess, and purification processes. Demonstrating similarity with respect to structure and in vitro biologic function and clinical PK is the foundation of establishing biosimilarity. Demonstration of clinical equivalence in efficacy, safety, and immunogenicity is an important step in confirming biosimilarity. The totality of evidence along with scientific justification based on a common mechanism of action, PK and clinical considerations in various patient populations is necessary to support extrapolation across indications.

ABP 215 is being developed as a biosimilar for treatment of the same indications as bevacizumab. Like bevacizumab, ABP 215 is produced by recombinant DNA technology in Chinese hamster ovary cells. Results from comprehensive analytical characterization studies have shown that ABP 215 and bevacizumab are physicochemically similar, notwithstanding minor differences that are not expected to affect the PK, safety, efficacy, and quality of the product. In addition, ABP 215 and bevacizumab (EU) have been shown to be functionally similar in an initial preclinical pharmacologic similarity assessment [12, 13]. Establishing similarity with respect to analytical and functional activity and PK parameters support a totality of biosimilarity along with further confirmation of efficacy and safety in patient studies. For biosimilars, Phase II dose-finding studies are neither necessary nor required by regulatory bodies.

This study is the first clinical study with ABP 215. As ABP 215 is being developed for use globally, this study was conducted at CPUs in the US and EU using regionally approved bevacizumab reference product as comparators as required by the biosimilar development pathway in each region. The results of this study demonstrate that the PK profile of ABP 215 is similar to both bevacizumab (US) and bevacizumab (EU). Moreover, bevacizumab (US) and bevacizumab (EU) were found to be bioequivalent to each other. Healthy male subjects were enrolled because healthy subjects provided the most sensitive population for assessing similarity of PK without the potentially confounding effects of disease states and concomitant drugs, which can alter PK profiles. Women were not included in this study of healthy volunteers because bevacizumab increases the risk of ovarian failure and may impair female fertility.

The standard criteria for demonstrating PK bioequivalence are that the two-sided 90% CI for the GMR must be within the prespecified acceptance range of 0.8 and 1.25 for overall exposure (e.g., AUC) [11, 12]. For each primary analysis comparison (AUC_inf_, AUC_last_, and *C*_max_), the 90% CIs for the adjusted least squares GMRs were fully contained within prespecified bioequivalence range. Bioequivalence between ABP 215 and bevacizumab (US) and bevacizumab (EU) was demonstrated and was consistent irrespective of the investigational site.

The safety, tolerability, and immunogenicity of ABP 215, bevacizumab (US), and bevacizumab (EU) were assessed by monitoring for AEs, clinical laboratory tests, vital signs measurements, ECGs, physical examinations, and by testing for the presence of ADA before administration of study medication and at EOS. All three study medications were safe and well tolerated. There were no deaths or SAEs, and no subjects discontinued the study as a result of AEs. In general, the safety profiles were similar across treatment groups. The majority of AEs were considered mild (grade 1) or moderate (grade 2) in intensity. Moreover, no new safety signals were identified beyond those expected based on previous studies and clinical experience with bevacizumab. In all three treatment arms, the most frequently reported AE considered possibly or probably related to study drug was headache. No clinically relevant changes in clinical laboratory tests, vital signs, ECGs, or physical examination findings were observed in any treatment group.

We further found that the incidence of AEs in the bevacizumab (EU) group (61%) was somewhat higher than in either the ABP 215 group (47%) or the bevacizumab (US) group (33%). In a post hoc summary of AEs, the incidence of any AE for the US site was 37.1% (13 subjects) in the ABP 215 group and 32.8% (22 subjects) in the bevacizumab (US) group; the incidence for the EU site was 57.6% (19 subjects) for ABP 215 and 61.2% (41 subjects) for bevacizumab (EU). Thus, although the frequencies of treatment-emergent AEs (TEAEs)s varied between the US and EU sites, the frequencies were similar between the ABP 215 and bevacizumab treatment groups within each individual site, further supporting the similarity between ABP 215 and bevacizumab.

Finally, bevacizumab reference product sourced from the (US) and from the (EU) were shown to be similar with respect to PK profiles, safety, and immunogenicity. Establishing similarity between reference products sourced from different regions is an important step in the developmental process for biosimilars. The US FDA and EMA have taken steps to facilitate global development programs by permitting the use of foreign-sourced comparators in Phase III clinical studies provided a “scientific bridge” between the local and the foreign-sourced reference product is demonstrated in comprehensive analytical similarity studies and PK study of the biosimilar candidate against both comparators. Thus, the scientific bridge between reference products allows for the use of single comparator in the Phase III clinical study. ABP 215 and bevacizumab were assessed in a Phase III study in patients with non-squamous non-small cell lung cancer. The results of the Phase III study will be reported in a separate communication that is currently in development.

The results of this Phase I study demonstrate PK bioequivalence of ABP 215, bevacizumab (US), and bevacizumab (EU). ABP 215, bevacizumab (US), and bevacizumab (EU) were safe and well tolerated. No new safety signals with regard to treatment with ABP 215 were identified and no subject tested positive for ADAs. The totality-of-evidence thus far indicates that ABP 215 is biosimilar to bevacizumab.
