# Correction to: A phase I, randomized, single-dose study evaluating the pharmacokinetic equivalence of biosimilar ABP 215 and bevacizumab in healthy adult men

**DOI:** 10.1007/s00280-017-3457-8

**Published:** 2017-11-20

**Authors:** Richard Markus, Vincent Chow, Zhiying Pan, Vladimir Hanes

**Affiliations:** Biosimilars Development, Amgen Inc., One Amgen Center Drive, Thousand Oaks, CA 91320 USA

## Correction to: Cancer Chemother Pharmacol (2017) 80:755–763 10.1007/s00280-017-3416-4

The article [A phase I, randomized, single-dose study evaluating the pharmacokinetic equivalence of biosimilar ABP 215 and bevacizumab in healthy adult men], written by [Richard Markus, Vincent Chow, Zhiying Pan and Vladimir Hanes], was originally published Online First without open access. After publication in volume [80], issue [4], page [755–763] the author decided to opt for Open Choice and to make the article an open access publication. Therefore, the copyright of the article has been changed to © The Author(s) [2017] and the article is forthwith distributed under the terms of the Creative Commons Attribution 4.0 International License (http://creativecommons.org/licenses/by/4.0/), which permits use, duplication, adaptation, distribution and reproduction in any medium or format, as long as you give appropriate credit to the original author(s) and the source, provide a link to the Creative Commons license, and indicate if changes were made. The original article was corrected.

